# A Process Related View on the Usage of Electronic Health Records from the Patients’ Perspective: A Systematic Review

**DOI:** 10.1007/s10916-022-01886-0

**Published:** 2022-12-29

**Authors:** Anna Griesser, Sonja Bidmon

**Affiliations:** Department of Marketing and International Management, Alpen-Adria-Universität Klagenfurt, Universitätsstraße 65-67, 9020 Klagenfurt am Wörthersee, Austria

**Keywords:** Electronic health record, Acceptance, Procedural view, Patient

## Abstract

**Background:**

In recent years, there has been an increasing interest in electronic health record (EHR) systems and various approaches of encouraging acceptance. Multiple methods of EHR acceptance have been proposed. However, a systematic review of patient's perspectives of their role and challenges in processing EHR remains lacking. Moreover, so far, there has been little discussion about barriers and facilitators of EHR system acceptance and usage from the patients' perspective.

**Methods:**

The study was reported according to the PRISMA statement. Six databases were systematically searched using keywords for articles from 2002–2020. We reviewed these data and used an inductive approach to analyse findings.

**Results:**

A total of 36 studies met the inclusion criteria. Our systematic literature review results reveal a wide range of barriers and facilitators assigned to four distinct stages of EHR system usage: awareness, adoption, behaviour and perception, and consequences. Results were described in a narrative synthesis of the included empirical studies.

**Discussion:**

Results underline the necessity to put a particular emphasis – but not exclusively – on the initial stage of awareness in the future. Further research in the field is therefore strongly recommended in order to develop tailored mediated communication to foster EHR system usage in the long run.

**Supplementary Information:**

The online version contains supplementary material available at 10.1007/s10916-022-01886-0.

## Introduction

A considerable amount of literature has been published on EHR platforms [1]. One of the earliest ideas and measures in the realm of health care at the beginning of digitalization in the early 1970s was the networking of patient-related electronic health records (EHR) between the necessary health care providers during treatment processes [2]. Basically stated, EHRs collect, archive, and administrate information on a patient's socio-demographic profile, vital signs, allergies, vaccinations, medical background and medication. The administration of this data relies on web-based applications, which can be controlled by the actors. Such platforms enable the continuous connection of patients, their health professionals, and other stakeholders (e.g., pharmacies, laboratories, insurance companies, care centres…) to present real patient data with all of the diseases, influencing factors, and distinctions that each individual patient has [1, 3, 4, 56]. The main objective of EHR platforms is the transparent exchange of information to ensure complete, efficient, and high-quality treatment [5, 6]. Nevertheless, so far patients’ acceptance of EHR is not satisfactory in most of the countries and regions all over the world. A large and growing body of literature has investigated acceptance of EHR from the health professionals’ perspective. To date, however, there has been little discussion in research with regard to EHR acceptance from the patients’ perspective [7]. Additionally, far too little attention has been paid to taking a procedural view on EHR acceptance and usage [8, 9]. Only a few papers aimed at examining the steps before or after the actual use of EHRs, and therefore shed light on different stages of the process of EHR usage. [10] Additionally, several authors relate “awareness” (e.g., [11–13]) to self-consciousness of patients. Although awareness was additionally considered, at least from time to time, in studies dealing with EHR acceptance and usage [5], the focus of the majority of EHR studies so far has been on the behaviour and perception stage as well as on the actual use of systems [3, 14–16]. Previous studies reveal that electronic health records have the potential to enhance patients’ commitment [1, 17, 18]. In practice, patients’ awareness of EHR systems can be increased through different marketing channels, for instance, public media, social media, etc. [18].

With regard to the range of theoretical foundations applied in EHR studies, the most widespread are ‘acceptance’ approaches including several factors which impede or facilitate individual or/and organizational decisions in adopting or accepting a technology: (1) The original *Technology Acceptance Model (TAM)* [19] developed by Davis, which conceptualizes perceived ease of use (PEOU) and perceived usefulness (PU) as central antecedents of technology acceptance, (2) the *Social Cognitive Theory (SCT)* [20] by Compeau and Higgins, proposing that individuals’ beliefs in self-efficacy influence actual behaviour; (3) and the *Unified Theory of Acceptance and Use of Technology (UTAUT) *[21] by Venkatesh et al. and the *UTAUT2* [22].

To the best of our knowledge, no study has taken a processual view on the acceptance and usage of EHR platforms so far. Similar concerns with regard to data privacy and the exchange of sensitive data, administered through web-based applications, exist in the area of mobile banking (mbanking) [3] or in other sensitive areas of the IT (Information and Technology) sector [23, 24]. Therefore, it might be fruitful to think outside the box and look at user acceptance of technologies in neighbouring technological areas, which have been well investigated with regard to user acceptance.

With the present study, we aimed to take a process-related view on patient use of EHR platforms in order to get a closer look at possible hurdles or saturated research areas. In the mobile banking sector, Larsen has developed a process-related view on mobile banking acceptance.

**Fig. 1:**
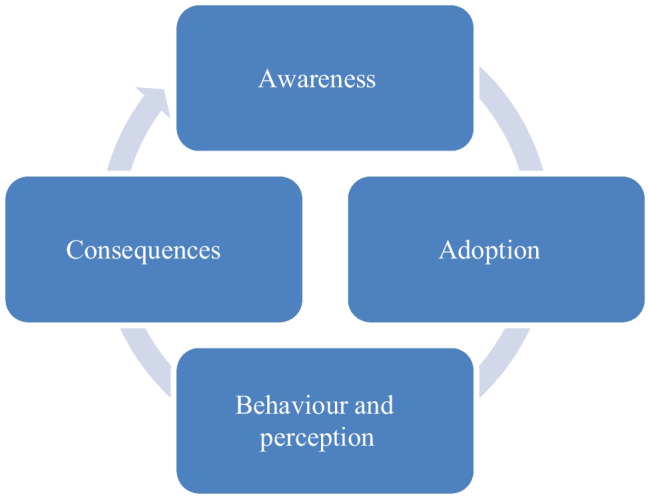
Stages of EHR Usage (Entire EHR Usage Process)

Figure 1 shows the process-related framework for our systematic literature review: From the first moment of awareness-raising (communication policy) [25] to adoption (expected usefulness, usability, risks) to the actual use of the platform (expected usefulness, usability, effectiveness) to the consequences (individual impact) that such use entails [26]. To this end, however, the additional prerequisite of ‘awareness’ in the entire influencing chain is going to be added in the current study. The step “awareness” is adapted at the individual level to express the perception of patients’ first sight of or/and actions with EHR [1]. In this review, we limit ourselves to a general patient view and not to the specific view of patients revealing specific disease patterns or medications. This more general view should allow us to generalize the results to a larger extent. Similarly, there is no preference for outpatient settings or inpatient settings in order to achieve a higher inclusion rate of studies found.

The research question for our systematic literature review is as follows: What are the barriers and facilitators of EHR acceptance and usage from a process-related view, i.e., in each of the steps of awareness (step 1), adoption (step 2), behaviour and perception (step 3) and consequences (step 4) included in empirical studies in the field so far?

## Methods

This study was reported according to the Preferred Reporting Items for Systematic Reviews and Meta-Analyses (PRISMA) statement [25]. This study aims to categorize different criteria along the entire influencing chain of the stages of 'awareness', 'adoption', 'behaviour and perception' and ‘consequences’ of EHR usage.

### Data Sources and Searches

The main inclusion criteria for studies were (1) focussing on the patients’ perspective (general/mixed patients; inpatients as well as outpatients) (2) when using EHRs (3) on web-based applications applying qualitative or quantitative approaches. The full literature search followed a traceable process in the following databases: PubMed, Science Direct, Cochraine Library, PsychINFO, Springer and additional articles by reference mining, as well as an additional simplified search in Google scholar to find public bodies and reference lists. The search strategy was applied equally in all databases. In order to generate search topics, the strategy selected search words, which were combined with 'OR' and the different search topics linked with 'AND'. Table 1 shows the applied search algorithm. All English and German articles in the period from 2001 to 2020 were eligible. There were no regional restrictions. An independent reviewer followed the entire literature process to achieve a final qualitative result.

**Table 1:** Search algorithm

**Search themes**			
	*(1) Patient perspective*	*(2) EHR*	*(3) web-based application*
**Keywords** (Applied across all databases)	‘patient’ ‘inpatient’ ‘outpatient’	‘electronic health records’ ‘EHR’ ‘health records’	‘portal’ ‘platform’

### Study Selection, Data Extraction and Quality Assessment

**Fig. 2:**
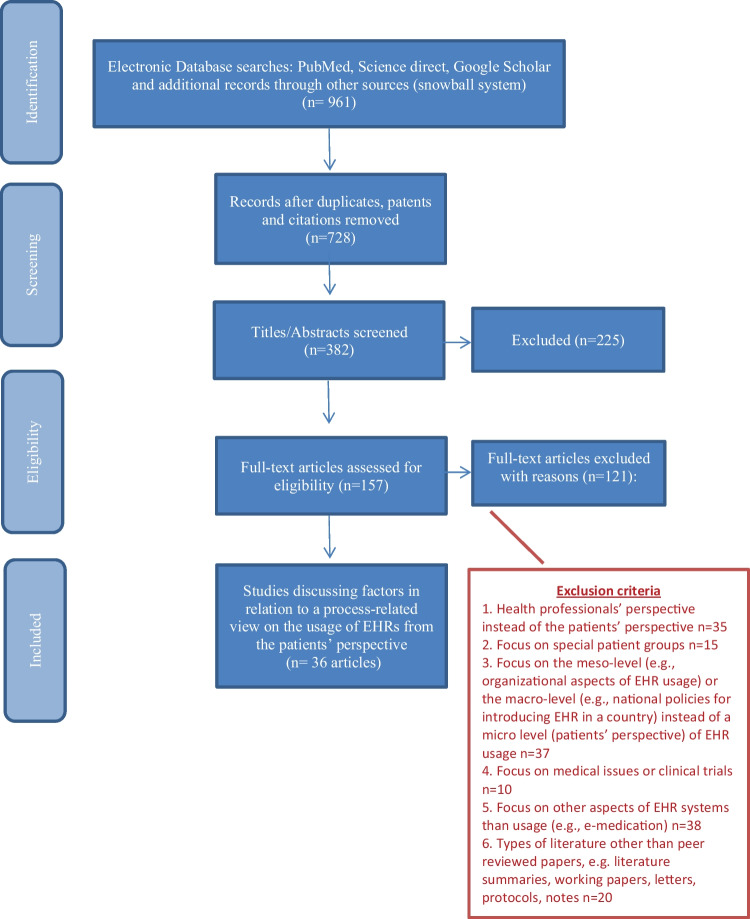
PRISMA Flow Diagram

As mentioned above, the entire process closely followed the guidelines of the PRISMA Statement to assess study quality and strength of evidence [25]. Figure 2 shows the four-step flow chart that describes the data extraction step by step. In addition, the multimedia appendix contains a 27-item checklist (Supplementary 2), which starts at the beginning of the review and runs through the entire study like a golden thread. The basic search process yielded a total of 961 results, of which 233 were duplicates, patents, or citations and these were therefore removed. The main author screened the remaining papers in detail by title/abstract to ensure their suitability for inclusion. Exclusions were made as follows (Table 2): Articles that focus specifically on (1) health professionals, (2) special patient groups, or (3) organizational aspects and national policies have been excluded. Also, (4) medical issues or clinical trials or articles solely discussing (5) differentiated views on EHRs or such systems (e.g., overall view, satisfaction issues, medication/prescription modules etc.) were excluded. Finally, (6) different types of papers dealing with an overview on literature in the field of interest found with our search algorithm (see Table 1) were excluded if the papers had another format than peer reviewed papers like, e.g., literature summaries not following a systematic literature review approach, working papers, letters, protocols, or notes. A total of 157 titles/abstracts met all defined inclusion criteria. The full-text articles meeting eligibility criteria were independently screened by two reviewers: the main author and an instructed Bachelor’s degree student (Cohen K = 0.94). The outstanding articles were reviewed and had to meet all inclusion criteria (Supplementary 1 – overview articles for eligibility). In cases of nonagreement (n = 9), studies were discussed, and consensus on inclusion or exclusion was reached by the team of authors [27]. In the end, 36 articles remained in the final corpus for the narrative synthesis.

**Table 2:** Exclusion criteria

Exclusion criteria*	***n***
1. Health professionals (doctor, nurse, other health staff)	35
2. Special patient groups (e.g. cancer, diabetes, cardiovascular, pneumo etc.)	15
3. Organizational (e.g. change process, IT-architecture etc.) or national aspects (government)	37
4. Medical issues or clinical trails	10
5. Differentiate view on electronic health records (e.g. overall view, satisfaction issues, medication/prescription modules etc.)	38
6. Literature summaries, working papers, letters, protocols, notes, i.e., papers not following a systematic literature review or a scientific searching approach like, e.g. the PRISMA statement	20

^*^Multiple answers per article possible

### Data Synthesis and Analytical Strategy

Table 3 shows the characteristics of the reviewed studies, disclosing authors and year, patient type (inpatient, outpatient, general/mixed), origin, title, method, and theory. The studies are referred consecutively with the linked number of the reference list. Subsequently, the content of articles was coded for allocation to three main categories (Table 1), which were inductively formed based on well-known frameworks [19–22]: (1) patient-related attributes, (2) social norm, and (3) technical/infrastructural attributes and assigned to the respective stage of the entire EHR usage process: awareness, adoption, behaviour and usage, consequences. Thus, all criteria were allocated within a 3 × 4 matrix with the “kind of attributes” and the stage of the usage process as the two axes. Again, the same two reviewers worked independently from each other and coded the variables included in the n = 36 studies analysed. All relevant psychographic attributes of patients together with social influences (social norm) and technical/infrastructural attributes, i.e., attributes inherent to the technology and the infrastructure used with regard to their EHR, were coded along the stages of EHR usage. The heterogeneity of the individual studies made the use of meta-analytical methods inappropriate for this review. Therefore, collected data were summarized by conducting a descriptive analysis and narrative synthesis, independently carried out by each of the two reviewers. The two reviewers scanned the papers independently from each other by applying a binary coding system (1 = variable was included in the respective study, 0 = variable was not included in the respective study). In cases of nonagreement, criteria/procedural steps were discussed by the authors, and a consensus was reached [27]. In the next step of the analysis, a conceptual model was developed based on the evaluated criteria in the studies (Tables 4, 5 and Supplementary 3).

**Table 3:** Summarized characteristics

**Characteristics**	***n***
Patient type	
• General/General/Mixed • Inpatient • Outpatient	22 8 6
Data collection method*	
• Survey • Systematic literature reviews, with a scientific searching approach reported by following e.g., the PRISMA statement • Interviews • Other approaches (e.g. observational studies, analytic models, focus groups…)	16 8 8 16
Empirical framework	
• Scientific theory • Self-constructed conceptual framework	6 30
Publication period	
• 2001 – 2005 • 2006 – 2010 • 2011 – 2015 • 2016—2020	4 2 12 18

^*^Multiple answers per article possible

**Table 4:** Characteristics General

**No**	**Author/** **Year**	**Patient type**	**Origin**	**Title**	**Method**	**Theory**	**No. references**
1	Moll, J. et al. (2018)	General/Mixed	Sweden	Patient's experiences of accessing their electronic health records: National patient survey in Sweden	Online survey	A conceptual framework (self-made)	[14]
2	Tavares, J. et al (2016)	General/Mixed	Portuguese	Electronic Health Record Patient Portal Adoption by Health Care Consumers: An Acceptance Model and Survey	Online questionnaire	Unified theory of acceptance and use of technology in a consumer context (UTAUT2)	[3]
3	Staroselsky, M. et al. (2005)	Out-patient	USA	Improving electronic health record (EHR) accuracy and increasing compliance with health maintenance clinical guidelines through patient access and input	Survey and control groups 6 months post survey	A conceptual framework (self-made)	[32]
4	Turner, K. et al. (2019)	General/Mixed	USA	Patient portal utilization: before and after stage 2 electronic health record meaningful use	Observational study	A conceptual framework (self-made)	[15]
5	Goel, M.S. et al. (2011)	General/Mixed	USA	Disparities in enrolment and use of an electronic patient portal	Observational, cross sectional study	A conceptual framework (self-made)	[28]
6	Hong, Y.A. et al. (2020)	General/Mixed	USA	Use of Patient Portals of Electronic Health Records Remains Low From 2014 to 2018: Results From a National Survey and Policy Implications	Logistic regression analysis	A conceptual framework (self-made)	[35]
7	Fraccaro, P. et al. (2018)	General/Mixed	Transnational	The influence of patient portals on users’ decision making is insufficiently investigated: A systematic methodological review	Systematic literature review	Coiera's information value chain	[47]
8	Pell, J.M. et al. (2015)	In-patient	USA	Patient Access to Electronic Health Records	Prospective cohort study	A conceptual framework (self-made)	[37]
9	Mold, F. et al. (2015)	Out-patient	Transnational	Patients’ online access to their electronic health records and linked online services: a systematic review in primary care	Systematic interpretative review	A conceptual framework (self-made)	[8]
10	Schwartz, P.H. et al. (2015)	Out-patient	USA	Patient Preferences in Controlling Access to Their Electronic Health Records: a Prospective Cohort Study in Primary Care	Survey	A conceptual framework (self-made)	[11]
11	Asan, O. et al. (2016)	In-patient	USA	Capturing the patients' voice: Planning for patient-centered electronic health record use	Semi-structured interviews + thematic analysis	A conceptual framework (self-made)	[30]
12	Zhao, J.Y. et al. (2018)	General/Mixed	Transnational	Barriers, Facilitators, and Solutions to Optimal Patient Portal and Personal Health Record Use: A Systematic Review of the Literature	Systematic literature review	A conceptual framework (self-made)	[40]
13	Huang, J. et al. (2019)	General/Mixed	USA	Difference Between Users and Nonusers of a Patient Portal in Health Behaviors and Outcomes: Retrospective Cohort Study	Retrospective observational cohort study	Novel cardinality matching approach	[46]
14	Dendere, R et al. (2019)	In-patient	Transnational	Patient Portals Facilitating Engagement With Inpatient Electronic Medical Records: A Systematic Review	Systematic literature review	A conceptual framework (self-made)	[34]
15	Munir, S. et al. (2001)	In-patient	USA	Patient empowerment and the electronic health record	Survey and interviews	A conceptual framework (self-made)	[53]
16	Woods, S.S. et al. (2017)	General/Mixed	USA	The Association of Patient Factors, Digital Access, and Online Behavior on Sustained Patient Portal Use: A Prospective Cohort of Enrolled Users	Prospective Cohort study	A conceptual framework (self-made)	[42]
17	Ancker, J.S. et al. (2015)	Out-patient	USA	Patient activation and use of an electronic patient portal	Survey	A conceptual framework (self-made)	[7]
18	Abd-alrazaq, A.A. et al. (2019)	General/Mixed	Transnational	Factors that affect the use of electronic personal health records among patients: A systematic review	Systematic literature review	Or and Karsh's conceptual framework	[18]
19	Shah, S. et al. (2015)	General/Mixed	United Kingdom	Accessing personal medical records online: A means to what ends?	Online survey questionnaire, and thematic analysis	A conceptual framework (self-made)	[10]
20	Mossaed, S. et al. (2015)	In-patient	Canada	Patient Preferences and Perspectives on Accessing Their Medical Records	Survey and observational study, with a thematic analysis	A conceptual framework (self-made)	[16]
21	Hoerbst, A. et al. (2010)	General/Mixed	Transnational	Attitudes and behaviors related to the introduction of electronic health records among Austrian and German citizens	Standardized interviews	A conceptual framework (self-made)	[12]
22	Greenhalgh, T. et al. (2008)	General/Mixed	United Kingdom	Patients' attitudes to the summary care record and HealthSpace: qualitative study	Semi-structured individual interviews and focus groups	A conceptual framework (self-made)	[13]
23	Zanaboni, P. et al. (2020)	General/Mixed	Norway	Patient Use and Experience With Online Access to Electronic Health Records in Norway: Results From an Online Survey	Online survey questionnaire	A conceptual framework (self-made)	[9]
24	Powell, K.R. (2017)	General/Mixed	Transnational	Patient-Perceived Facilitators of and Barriers to Electronic Portal Use: A Systematic Review	Systematic literature review	A conceptual framework (self-made)	[1]
25	Huvila, I. et al. (2015)	General/Mixed	Sweden	Patients' Perceptions of their medical records form different subject positions	Survey questionnaire	A conceptual framework (self-made)	[48]
26	Goldzweig, C.L. et al. (2013)	General/Mixed	Transnational	Electronic Patient Portals: Evidence on Health Outcomes, Satisfaction, Efficiency, and Attitudes: A Systematic Review	Systematic literature review	A conceptual framework (self-made)	[31]
27	Alpert, J.M. et al. (2016)	General/Mixed	USA	Applying Multiple Methods to Comprehensively Evaluate a Patient Portal's Effectiveness to Convey Information to Patients	Interviews, focus groups and thematic content analysis	A conceptual framework (self-made)	[29]
28	Hanna, L. et al. (2017)	General/Mixed	Australia	Patient perspectives on a personally controlled electronic health record used in regional Australia	Semi-structured telephone interviews, and inductive analysis	A conceptual framework (self-made)	[41]
29	Zarcadoolas, C. et al. (2013)	General/Mixed	USA	Consumers' perceptions of patient-accessible electronic medical records	Focus groups	Grounded theory	[50]
30	Goel, M.S. et al. (2011)	In-patient	USA	Patient reported barriers to enrolling in a patient portal	Telephone survey questionnaire	A conceptual framework (self-made)	[33]
31	Honeyman, A. et al. (2005)	Out-patient	United Kingdom	Potential impacts of patient access to their electronic care records	Semi-structured prospective interviews	A conceptual framework (self-made)	[51]
32	Pyper, C. et al. (2004)	Out-patient	United Kingdom	Patients' experiences when accessing their online electronic patient records in primary care	In-depth interviews using semi-structured questionnaires and a series of focus group	A conceptual framework (self-made)	[39]
33	Wass, S. et al. (2017)	General/Mixed	Sweden	Exploring patients' perceptions of accessing electronic health records: Innovation in healthcare	Inpatient interviews and outpatient surveys	A conceptual framework (self-made)	[36]
34	van Mens, H.J.T. et al. (2019)	General/Mixed	Transnational	Determinants and outcomes of patient access to medical records: Systematic review of systematic reviews	Systematic literature review	Clinical adoption framework (CFA)	[43]
35	Nambisan, P. et al. (2017)	In-patient	USA	Factors that impact Patient Web Portal Readiness (PWPR) among the underserved	Survey	A conceptual framework (self-made)	[38]
36	Woods, S.S. et al. (2013)	In-patient	USA	Patient experiences with full electronic access to health records and clinical notes through the My HealtheVet Personal Health Record Pilot: qualitative study	Focus group sessions	A conceptual framework (self-made)	[54]

**Table 5:** Catalogue of criteria

	**Patient-related attributes**				**Social norm**	**Technical/Infrastructural attributes**
	**Sociodemographic factors**	**Psychological factors**		**Health-related factors**		
**Step**		*Activating*	*Cognitive*			
Awareness	22		10; 17; 18; 21; 22; 24	12; 20; 21; 22		2; 11; 20; 21; 22
Adaption	30; 34	30	2; 3; 14; 26; 30; 34	14; 30	2; 30	14; 30
Behavior and perception	1; 2; 4; 5; 6; 8; 9; 10; 11; 12;; 13; 15; 16; 17; 18; 20; 21; 23; 24; 26; 29; 31; 33; 35	12; 15; 25; 31	1; 2; 4; 6; 7; 8; 9; 10; 11; 12; 13; 16; 17; 18; 19; 20; 21; 23; 24; 25; 26; 27; 28; 29; 31; 32; 33; 35	2; 3; 6; 9; 11; 12; 13; 15; 23; 24; 25; 26; 27; 28; 29; 31; 32; 33; 35	12	1; 3; 6; 7; 8; 9; 10; 11; 12; 15; 16; 17; 18; 20; 23; 24; 25; 27; 28; 29; 31; 32; 33; 35
Consequences		2	1; 2; 3; 7; 12; 19; 33; 36	3; 19; 36		19; 33; 36

The majority of studies included general/mixed (n = 22), then outpatient (n = 6) and inpatient (n = 8) patients. Most commonly, surveys (n = 16) were used as data collection method, and a minority of studies applied other approaches, for instance, observational studies, analytic models, or focus groups (n = 16).

Studies were mostly based on self-constructed conceptual frameworks (n = 30); only six studies applied a scientifically accepted theory. With regard to the publication period, it can be seen that the topic gained interest over the years, starting with only n = 2 papers between 2001 and 2005. As can be seen in Table 3, there has been a disproportionate increase in the number of papers dealing with EHR usage over the past twenty years.

### Awareness of EHR Systems (n = 10)

#### Patient-related Attributes

Six studies dealt with the patient's cognitive expectations, i.e., involving the patient to manage his or her personal data should increase the understanding of the system from the very beginning [13, 18]. Nevertheless, patients’ empowerment, through (a) know-how, (b) self-efficacy or (c) commitment showed low assurance in the studies, and (d) patients’ motivation to adopt a system was crucial. The patients' lack of information or confidence, or a purely negative attitude (e.g., lack of usefulness, complexity of the system) contributed to the failure of the system’s adoption [12, 28, 55]. The most important leverage points to raise awareness were the implementation of actions such as (individual) log-in training, workshops on navigation and handling in and with the system, etc. Patients whose providers communicated and encouraged them to use the portal in a first step perceived this as their main stimulus, leading to an increased awareness [1, 18, 29, 57]. Health determinants (n = 4) such as chronic pre-existing conditions or acute complaints were decisive factors that could impede awareness of EHRs.

### Technical/Infrastructural Attributes

Technical/infrastructural conditions were similarly pronounced (n = 5). Results highlighted the patients’ requirement for appropriate technical equipment for the use of EHR [3, 28]. The presentation of the possible structure and navigation of the EHR system, as well as features, may strongly limit the patients’ awareness in advance. Also, factors such as information security and privacy, with possible restrictions/regulations by the patients of their sensitive data were strongly addressed in the first step of awareness-raising [30]. The ignorance of legal rights by patients also limited the adoption of EHR systems [16].

### Adoption of EHR Systems (n = 6)

#### Patient-related Attributes

Studies show that a digital divide in our society still seems to exist thus adoption varies due to age, gender or cultural affiliation. Also, the patients’ subjective attitude could control the use of such systems, by means of someone’s expectations, individual self-perceptions, or habits (n = 6) [31, 32]. With regard to health-related factors in the stage of adoption, two studies have dealt more intensively with the effects on the health outcome and on the implementation or acceptance of medical recommendations. Such attributes represented obstacles on the one hand, but also encouraging factors in the course of EHR adoption [13].

#### Social Norm

Due to social influences [3, 33] such as family, friends, etc., patients were more or less inclined to use electronic health data or not. Group formations relating to similar previous illnesses or complaints seemed to be additionally relevant in this vein.

#### Technical/Infrastructural Attributes

Two studies [33, 34] took a closer look at the technical/infrastructural factors, which consist of all design-related aspects, including surface, content, features, and functions. A satisfactory design seems to be an essential facilitator during this stage. The results show that the barriers in this stage were unclear information security (e.g., the internet as an unsafe way to communicate) and regulations regarding access to personal health data (e.g., restricted access to EHRs for patients) [33].

### Behaviour and Perception of EHR Systems (n = 29)

#### Patient-related Attributes

A total of 24 studies dealt with socio-demographic attributes such as age, gender, race, income, insurance status, education, etc. in relation to the use of electronic health data. Also, patients with a certain degree of previous experience due to past actions have a significant facilitating influence on the use of such systems [35, 36]. Additionally, patients’ intrinsic motivation seemed to be the most prominent activating factor when it comes to using an EHR system [9, 37, 53]. In contrast, patients’ existing concerns or barriers range from personal attributes (i.e., a lack of perceived usefulness, and familiarity with digital technologies, concern with regard to information security, too little know-how in handling digital devices, etc.), to technical/infrastructural (i.e., insufficient equipment, poor internet access, etc.) barriers. Furthermore, chronic pre-existing conditions, with factors such as the complexity and duration of illness, may lead to a differentiated use of the system by patients, as the focus, as well as the interest for self-benefit, are set differently [1, 38, 39, 55].

#### Social Norm

One study demonstrated that social influence (e.g., family, friends, health staff) was essential, especially among older patients who often require assistance when using the platform. Also, during registration, there is a steady need for assistance, at least in the target group of older patients [40].

### Technical/Infrastructural Attributes

Technical and infrastructural factors constituted important facilitators or barriers at the stage of use (n = 24). A system that is easy to understand and tailored to patient needs increased the chances of a high level of use of the system [18, 33, 38, 40]. A clear information and communication policy adapted to the user group was therefore essential to ensure transparent communication [41, 42]. Concerns with regard to information security and privacy were also important barriers. Based on legal data policies, secure data transfer in relation to health professionals, as well as of the systems, seem to be essential ingredients of a satisfactory usage of EHR nowadays [1, 12, 13].

### Consequences of EHR System Usage (n = 8)

#### Patient-related Attributes

In the step of the consequences of the EHR system usage, patient empowerment (i.e., checking and monitoring one’s health status) and activation (i.e., learning medical terms or use of digital technologies) revealed the highest importance [11, 40, 54]. Furthermore, the increased physician–patient contact during (i.e., messenger, notifications, etc.) and after treatment (i.e., requirements regarding reports, medical diagnoses, prescription renewals, appointment reminders, etc.) between health professionals and patients strengthened the positive impact of EHR systems. Also, further patient adherence and utilization (i.e., in case of uncertainties regarding laboratory results, discrepancies in medication, etc.) have to be considered [10, 32, 43, 54, 55].

#### Technical/Infrastructural Attributes

Little information was found on technical/infrastructural attributes (n = 3). In principle, however, gaps were identified that restrict proper use. Barriers arose due to access regulations with regard to the system, log-in difficulties, less intuitive navigation, non-existent information or simply the lack of the right equipment [10, 36, 42].

#### Discussion

The present systematic literature review identified 36 studies that give valuable insight into the barriers and facilitators of EHR use. Moreover, the identified facilitators and barriers were assigned to different stages by taking a procedural view on the acceptance and usage of EHR systems [4, 44, 45]. Strongly reported in the studies, over the first three procedural steps, were (a) socio-demographic factors (i.e., age, gender, ethnicity, education level, or income) such as higher health consciousness in women than in men or poor competencies in handling EHR systems in older people, or in terms of cultural background such as among Latinos and Blacks [16, 46]. (b) Psychological-cognitive factors occur in all four steps (i.e. frequent internet usage or online health-related information searching lead to a better handling of an EHR system [34, 47, 55], patient empowerment and activation enables patients to take an active part in the digital treatment process [7, 48], and patient education and training as the main facilitator in the beginning and also continuing to ensure smooth handling by the patients) [1, 8]; as well as (c) health-related factors (i.e. patient-specific systems, guidelines or treatment plans relating to their health status, e.g., in case of chronic disability) [6, 49]; and (d) technological/infrastructural attributes (i.e., good surface design, structured and safe information regarding diagnosis, medication or prescription, or easy and private communication pathways, with specific information regarding the treatment process or direct digital contacts with provider) [47, 50], while low results occur over all procedural steps for (e) social influence of family and peer members (i.e. need for assistance during registration and also usage) [3, 33] and (f) psychological-activating factors (i.e. individual negative attitude or solely no interest toward new technologies) [51]. Nevertheless, it appears that patients’ EHR confidence levels fluctuate along the process-related view of usage [5, 13, 52]. Results revealed the importance of ensuring that handling EHRs is included in the first step of awareness raising. With the help of measures through various communication channels, EHRs can be made more appropriate for patients [5]. Improving patients’ awareness of EHRs is critical before an applied system is recommended [13, 34].

### Limitations

Although we applied a thorough search strategy and conducted a diligent reprocessing of the studies we included, studies for special patient groups (e.g., cancer, diabetes, etc.), as well as studies exploring the perspective of health professionals were excluded. Furthermore, this review focused predominantly on broad research trends and gaps from the procedural view of patient use. Thus, future studies will need to assess in greater depth any research gaps such as patients’ skill awareness and final performance using a more diverse range of survey instruments.

## Conclusions

By taking a procedural view on acceptance and use of EHR systems, the present systematic literature review has identified several facilitators and barriers along the different stages of the process of using EHR systems. In a next step, a qualitative approach has to be taken to investigate the patients’ perspectives on possible barriers and facilitators in order to evaluate the results of the systematic literature review from a practical point of view. The results of the subsequent qualitative approach (patients’ view) can then be used to develop communication strategies and tools for their practical implementation in the form of tailored mediated health communication. In the light of the present pandemic, an increase in the usage of EHR systems could be essential for different stakeholders. From a macro perspective (i.e., the health care system), an increased usage of EHR systems could contribute to justify the enormous (sunk) costs incurred for the ambitious roll-out plans in many countries all over the world. From a meso perspective (i.e., the level of health providers’ organisations like hospitals), an increased EHR usage can boost the usefulness of information exchange on EHR platforms and deliver a raison d'être in the long run. From the micro perspective (i.e., the personal level of patients), which was the focus of the present study, several facilitators and barriers could be identified. Being creative in developing innovative and useful features of EHR systems like, e.g., integrating the vaccination status and generating automatic reminders for having a booster shot, could also encourage usage of EHR systems in the long run on the personal level. A profound knowledge of the levers for increasing the use of EHR could be used in a next step to develop mediated health communication targeted at raising the awareness of EHR and bringing the patients further along the entire usage process. Changes on the micro level, could then, lead to changes on the meso and the macro level as well, at least in the long run.

### Supplementary Information

Below is the link to the electronic supplementary material.

Supplementary file1 (XLSX 32 KB)

Supplementary file2 (DOCX 34 KB)

Supplementary file3 (XLSX 15 KB)

## Data Availability

The authors confirm that the data supporting the findings of this study are available within the article [and/or] its supplementary materials.

## References

[1] Powell, K.R. Patient-Perceived Facilitators of and Barriers to Electronic Portal Use: A Systematic Review. CIN: Computers, Informatics, Nursing. **35**, 565–573 (2017). 10.1097/CIN.0000000000000377 28723832

[2] Meister, S, Deiters ,W. & Becker, S. Digital health and digital biomarkers – enabling value chains on health data. Current Directions in Biomedical Engineering. **2**, 577–581 (2016).

[3] Tavares, J. & Oliveira, T. Electronic health record patient portal adoption by health care consumers: An acceptance model and survey. Journal of Medical Internet Research. **18** (2016). 10.2196/jmir.5069 PMC4795321 26935646

[4] Evans RS (2016). Electronic Health Records: Then, Now, and in the Future. Yearb Med Inform..

[5] Riordan F (2015). Patient and public attitudes towards informed consent models and levels of awareness of Electronic Health Records in the UK. International Journal of Medical Informatics..

[6] Menachemi N, Collum TH (2011). Benefits and drawbacks of electronic health record systems. Risk Manag Healthc Policy..

[7] Ancker JS (2015). Patient activation and use of an electronic patient portal. Inform Health Soc Care..

[8] Mold F, de Lusignan S, Sheikh A (2015). Patients’ online access to their electronic health records and linked online services: a systematic review in primary care. Br J Gen Pract..

[9] Zanaboni P, Kummervold PE, Sorensen T, Johansen MA (2020). Patient Use and Experience With Online Access to Electronic Health Records in Norway: Results From an Online Survey. J Med Internet Res..

[10] Shah SGS (2015). Accessing personal medical records online: a means to what ends?. Int J Med Inform..

[11] Schwartz PH (2015). Patient preferences in controlling access to their electronic health records: a prospective cohort study in primary care. J Gen Intern Med..

[12] Hoerbst A, Kohl CD, Knaup P, Ammenwerth E (2010). Attitudes and behaviors related to the introduction of electronic health records among Austrian and German citizens. Int J Med Inform..

[13] Greenhalgh T, Wood GW, Bratan T, Stramer K, Hinder S (2008). Patients’ attitudes to the summary care record and HealthSpace: qualitative study. BMJ..

[14] Moll J (2018). Patients’ Experiences of Accessing Their Electronic Health Records: National Patient Survey in Sweden. J Med Internet Res..

[15] Turner K, Hong YR, Yadav S, Huo J, Mainous AG (2019). Patient portal utilization: before and after stage 2 electronic health record meaningful use. Journal of the American Medical Informatics Association..

[16] Mossaed S, Leonard K, Eysenbach G (2015). Patient Preferences and Perspectives on Accessing Their Medical Records. Journal of Medical Imaging and Radiation Sciences..

[17] Wiljer D (2008). Patient accessible electronic health records: exploring recommendations for successful implementation strategies. J Med Internet Res..

[18] Abd-Alrazaq AA, Bewick BM, Farragher T, Gardner P (2019). Factors that affect the use of electronic personal health records among patients: A systematic review. Int J Med Inform..

[19] Davis FD (1989). Perceived Usefulness, Perceived Ease of Use, and User Acceptance of Information Technology. MIS Quarterly..

[20] Compeau DR, Higgins CA (1995). Computer Self-Efficacy: Development of a Measure and Initial Test. MIS Quarterly..

[21] Venkatesh V, Morris MG, Davis GB, Davis FD (2003). User Acceptance of Information Technology: Toward a Unified View. MIS Quarterly..

[22] Venkatesh V, Thong XU (2012). Consumer Acceptance and Use of Information Technology: Extending the Unified Theory of Acceptance and Use of Technology. MIS Quarterly..

[23] DeLone WH, McLean ER (1992). Information Systems Success: The Quest for the Dependent Variable. Information Systems Research..

[24] DeLone, W.H. & McLean, E.R. Information systems success revisited. Proceedings of the 35th Annual Hawaii International Conference on System Sciences. IEEE Comput. Soc. 2966–2976 (2002).

[25] Moher, D. Preferred Reporting Items for Systematic Reviews and Meta-Analyses: The PRISMA Statement. Ann Intern Med. 151–264 (2009). 10.7326/0003-4819-151-4-200908180-00135 19622511

[26] Larsen KRT (2003). A Taxonomy of Antecedents of Information Systems Success: Variable Analysis Studies. Journal of Management Information Systems..

[27] Landis, J.R & Koch, G.G. The measurement of observer agreement for categorical data. Biometrics. **33**, 159–1741 (1977). 843571

[28] Goel MS (2011). Disparities in Enrollment and Use of an Electronic Patient Portal. J Gen Intern Med..

[29] Alpert JM, Krist AH, Aycock RA, Kreps GL (2016). Applying Multiple Methods to Comprehensively Evaluate a Patient Portal’s Effectiveness to Convey Information to Patients. J Med Internet Res..

[30] Asan O, Tyszka J, Fletcher KE (2016). Capturing the patients’ voices: Planning for patient-centered electronic health record use. International Journal of Medical Informatic..

[31] Goldzweig CL (2013). Electronic patient portals: evidence on health outcomes, satisfaction, efficiency, and attitudes: a systematic review. Ann Intern Med..

[32] Staroselsky M (2006). Improving electronic health record (EHR) accuracy and increasing compliance with health maintenance clinical guidelines through patient access and input. International Journal of Medical Informatics..

[33] Goel MS (2011). Patient reported barriers to enrolling in a patient portal. J Am Med Inform Assoc..

[34] Dendere R (2019). Patient Portals Facilitating Engagement With Inpatient Electronic Medical Records: A Systematic Review. J Med Internet Res.

[35] Hong, Y.A., Jiang, S. & Liu, P.L. Use of Patient Portals of Electronic Health Records Remains Low From 2014 to 2018: Results From a National Survey and Policy Implications. Am J Health Promot. **34**, 677–680 (202). 10.1177/0890117119900591 32030989

[36] Wass S, Vimarlund V, Ros A (2019). Exploring patients’ perceptions of accessing electronic health records: Innovation in healthcare. Health Informatics J..

[37] Pell JM, Mancuso M, Limon S, Oman K, Lin CT (2015). Patient Access to Electronic Health Records During Hospitalization. JAMA Intern Med..

[38] Nambisan P (2017). Factors that impact Patient Web Portal Readiness (PWPR) among the underserved. International Journal of Medical Informatics..

[39] Pyper C, Amery J, Watson M, Crook C (2004). Patients’ experiences when accessing their on-line electronic patient records in primary care. Br J Gen Pract..

[40] Zhao, J.Y., et al. Barriers, Facilitators, and Solutions to Optimal Patient Portal and Personal Health Record Use: A Systematic Review of the Literature. AMIA Annu Symp Proc, 1913–1922 (2017). PMC5977619 29854263

[41] Hanna L, Gill SD, Newstead L, Hawkins M, Osborne RH (2017). Patient perspectives on a personally controlled electronic health record used in regional Australia: ‘I can be like my own doctor’. HIM J..

[42] Woods SS (2017). The Association of Patient Factors, Digital Access, and Online Behavior on Sustained Patient Portal Use: A Prospective Cohort of Enrolled Users. J Med Internet Res..

[43] van Mens HJT, Duijm RD, Nienhuis R, de Keizer NF, Cornet R (2019). Determinants and outcomes of patient access to medical records: Systematic review of systematic reviews. International Journal of Medical Informatics..

[44] OECD. Key Issues for Digital Transformation in the G20. Available at: http://www.oecd.org/G20/key-issues-for-digital-transformation-in-the-G20.pdf?utm_source=Adestra&utm_medium=email&utm_content=Read%20the%20OECD%27s%20report%20for%20the%20G20%E2%80%A6&utm_campaign=OECD%20Science%2C%20Technology%20and%20Innovation%20News%20Jan17&utm_term=demo (2022).

[45] Kim, E.H., Wang M., Lau, C. & Kim, Y. Application and Evaluation of Personal Health Information Management System. Conf Proc IEEE Eng Med Biol Soc. 3159–3162 (2004). 10.1109/IEMBS.2004.1403891 17270950

[46] Huang J, Chen Y, Landis JR, Mahoney KB (2019). Difference Between Users and Nonusers of a Patient Portal in Health Behaviors and Outcomes: Retrospective Cohort Study. J Med Internet Res..

[47] Fraccaro P (2018). The influence of patient portals on users’ decision making is insufficiently investigated: A systematic methodological review. International Journal of Medical Informatics..

[48] Huvila I, Cajander A, Daniels M, Ahlfeldt RM (2015). Patients’ perceptions of their medical records from different subject positions. J Assn Inf Sci Tec.

[49] Cowell C (2002). Findings from the South Staffordshire opt-out patient consent campaign. Health Expect..

[50] Zarcadoolas C, Vaughon WL, Czaja SJ, Levy J, Rockoff ML (2013). Consumers’ perceptions of patient-accessible electronic medical records. J Med Internet Res..

[51] Honeyman A, Cox B, Fisher B (2005). Potential impacts of patient access to their electronic care records. Inform Prim Care..

[52] WHO. A health telematics policy in support of WHO’s Health-for-all strategy for global health development. Available at: https://apps.who.int/iris/bitstream/handle/10665/63857/WHO_DGO_98.1.pdf?sequence=1&isAllowed=y (2022).

[53] Munir S, Boaden R (2001). Patient empowerment and the electronic health record. Stud Health Technol Inform..

[54] Woods SS (2013). Patient experience with full electronic access to health records and clinical notes through the My HealthVet Personal Health Record Pilot: qualitative study. J Med Internet Res..

[55] Holderried M (2021). Attitude and potential benefits of modern information and communication technology use and telemedicine in cross-sectoral solid organ transplant care. Sci Rep.

[56] Rotmensch M, Halpern Y, Tlimat A, Horng S, Sontag D (2017). Learning a Health Knowledge Graph from Electronic Medical Records. Sci Rep.

[57] Gensheimer SG (2018). Oh, the places we’ll go: Patient-reported outcomes and electronic health records. Patient.

